# Does craniofacial morphology affect third molars impaction? Results from a population-based study in northeastern Germany

**DOI:** 10.1371/journal.pone.0225444

**Published:** 2019-11-22

**Authors:** Stefan Kindler, Till Ittermann, Robin Bülow, Birte Holtfreter, Catharina Klausenitz, Philine Metelmann, Maria Mksoud, Christiane Pink, Christian Seebauer, Thomas Kocher, Thomas Koppe, Karl-Friedrich Krey, Hans-Robert Metelmann, Henry Völzke, Amro Daboul

**Affiliations:** 1 Department of Oral and Maxillofacial Surgery/Plastic Surgery, University Medicine Greifswald, Greifswald, Germany; 2 Institute for Community Medicine, University Medicine Greifswald, Greifswald, Germany; 3 Institute for Diagnostic Radiology and Neuroradiology, University Medicine Greifswald, Greifswald, Germany; 4 Department of Restorative Dentistry, Periodontology, Endodontology, and Preventive and Pediatric Dentistry, University Medicine Greifswald, Greifswald, Germany; 5 Department of Orthodontics, University Medicine Greifswald, Greifswald, Germany; 6 Department of Anatomy and Cell Biology, University Medicine Greifswald, Greifswald, Germany; 7 Department of Prosthodontics, Gerodontology and Biomaterials, University Medicine Greifswald, Greifswald, Germany; Thamar University, Faculty of Dentistry, YEMEN

## Abstract

**Objectives:**

It is still not clear why impaction of third molars occurs. Craniofacial morphology and facial parameters have been discussed to be strong predictors for third molar impaction. Thus, this study aimed to investigate the effect of craniofacial morphology on erupted or impacted third molars in a German population sample.

**Materials and methods:**

Erupted and impacted third molars in 2,484 participants from the Study of Health in Pomerania were assessed by whole-body magnetic resonance imaging. Markers of facial morphology were determined in 619 individuals of those participants in whose 421 participants (16.7%) had at least one impacted third molar. Craniofacial morphology was estimated as linear measurements and was associated in a cross-sectional study design with impacted and erupted third molars by multinomial logistic regression models. Erupted third molars were used as reference outcome category and regression models were adjusted for age and sex.

**Results:**

Maximum Cranial Width (Eurion-Eurion distance) was significantly associated with impacted third molars (RR: 1.079; 95% confidence interval 1.028–1.132). This association was even more pronounced in the mandible. Individuals with a lower total anterior facial height (Nasion-Menton distance) and a lower facial index also have an increased risk for impacted third molars in the mandible (RR 0.953; 95% confidence interval 0.913–0.996 and RR: 0.943; 95% confidence interval 0.894–0.995). No significant associations of third molar status with facial width (Zygion-Zygion distance), and sagittal cranial dimension (Nasion-Sella distance; Sella-Basion distance) were observed.

**Conclusion:**

Individuals with an increased maximal cranial width have a higher risk for impaction of third molars in the mandible and in the maxilla. Individuals with a lower anterior total anterior facial height and lower facial index also have an increased risk for third molars impaction in the mandible. These findings could help orthodontic dentists, oral surgeons and oral and maxillofacial surgeons in decision-making for third molars removal in their treatment. These findings highlight the necessity of an additional analysis of the maximal cranial width by the Eurion- Eurion distance.

## Introduction

Third molars are the most frequent impacted human teeth and their removal is a frequent surgical procedure in dentistry [1, 2]. Nevertheless, it is still not clear what causes the impaction of third molars [3].

It is hypothesized that during human evolution the jaw size has decreased more rapidly than the size of teeth and therefore, an increased impaction of third molars as last erupting teeth occurred [4–6]. A similar observation was noted in craniodental allometric analysis of monkeys, where smaller craniums and jaw sizes were discussed to lead to third molars being crowded out of the jaws into an evolutionary loss [4]. This crowding out was associated with a shortening of the face and the mandible [7]. Even though dental eruption sequence may also be conserved phylogenetically in primates, it has been suggested that the dental eruption sequence could also be related to body and brain size. [8, 9] Facial parameters like face height and face width are discussed as predictors for third molar impaction in humans [3, 10].

Moreover, variations in craniofacial morphology may explain different impaction proportions of third molars worldwide. In Africa the lowest prevalence of impacted third molars is described [3], whereas the occurrence of impacted third molars is higher in the Middle East than in Asia [3, 11–13]. A skeletal facial type providing increased space is hypothesized for full eruption of third molars[10]. Brachyfacial individuals have a short anterior facial height and a wide face, whereas dolichofacial individuals have a long anterior facial height and a narrow face [10, 14]. However, inconsistencies and contradictions in the literature investigating the association of craniofacial morphology and third molars impaction exist. In a total of 98 orthodontic patients of the Royal Dental Hospital of Melbourne who received orthopantomograms and lateral cephalometric radiographs, respectively a higher prevalence of impacted third molars was reported in dolichofacial individuals than in brachyfacial patients [10]. In contrast, in 162 Argentinian patients and in 50 Italian patients who received cephalometric radiographs a higher prevalence of impacted third molars in brachycephalic individuals, than in dolichofacial individuals was described. [15, 16] These contradicting results may be due to selection bias caused by a necessity to avoid unnecessary radiograpic diagnostics [3] or due to limitations of 2D assessment modalities. Assessment of craniofacial morphology in orthodontic patients is normally conducted with orthopantomograms, photometric analysis and cephalometric radiographs [17, 18]. Assessment of craniofacial morphology with these imaging modalities may have limitations [17, 19] and landmark detection of the craniofacial complex with 3D imaging could allow a more advanced evaluation than with 2D imaging [20].

Magnetic resonance imaging (MRI) is a non-invasive 3D imaging modality without X-ray exposure and MRI is established in head imaging [21]. MRI has been used for assessment of head and neck cancer [22], for diagnosis of impacted third molars [23], for diagnosis of sinusitis [24], for evaluation of the temporomandibular joint, for implant planning [25] and for analyzing craniofacial structures [25–29]. MRI is also suitable for three-dimensional measurements of the craniofacial skeleton and for analysis of craniofacial morphology which is difficult to describe with cephalometric radiography [26, 30]. Different studies analyzing the craniofacial morphology have used MRI to achieve reliable and reproducible measurements [31–33]. In a population-based study in the Northeast of Germany a whole-body MRI research project was embedded aiming to identify risk factors, subclinical disease in terms of prevention and health care strategies [29].

Correlations between craniofacial morphology and third molars can be clinically relevant for planning and assessment of orthodontic and combined orthodontic and maxillofacial surgery treatment [34, 35]. Among orthodontic clinicians the importance of third molars for relapse after orthodontic therapy is controversially discussed and a great variation regarding their indication for removal exists [36, 37]. Three dimensional cephalometric analysis of craniofacial structures may be useful for assessment of orthodontic therapy [38, 39] and also may be helpful in clinical decision-making for removal of impacted third molars[40].

This study is a hypothesis generating study that aimed to clarify associations of MRI-derived craniofacial morphology distances with MRI-derived third molar status in a Caucasian descent population.

## Material and methods

### Study population

The Study of Health in Pomerania (SHIP) is a population-based study in the Northeast of Germany [41]. For baseline SHIP (1997–2001), a random cluster sample was collected [42]. For the second examination follow-up (SHIP-2) from 2008–2012 3,708 eligible individuals were re-invited, of which 2,333 participated. The local Ethics Committee of the University of Greifswald approved study protocols and written informed consent was obtained from each participant. All examinations were performed in accordance with the Declaration of Helsinki.

The present study is based on data of 2,333 participants from SHIP-2 (n = 2,333) Whole body MRI was performed in 1,115 individuals. Of those, 832 participants had erupted or impacted third molars and 213 were not available for craniofacial morphology. Of the 1.115 individuals with an MRI scan, 619 individuals were selected for this study after excluding MRI images with artifacts and pathologies affecting the craniofacial area of interest (S1 Table).

### Third molars imaging

**Fig 1:**
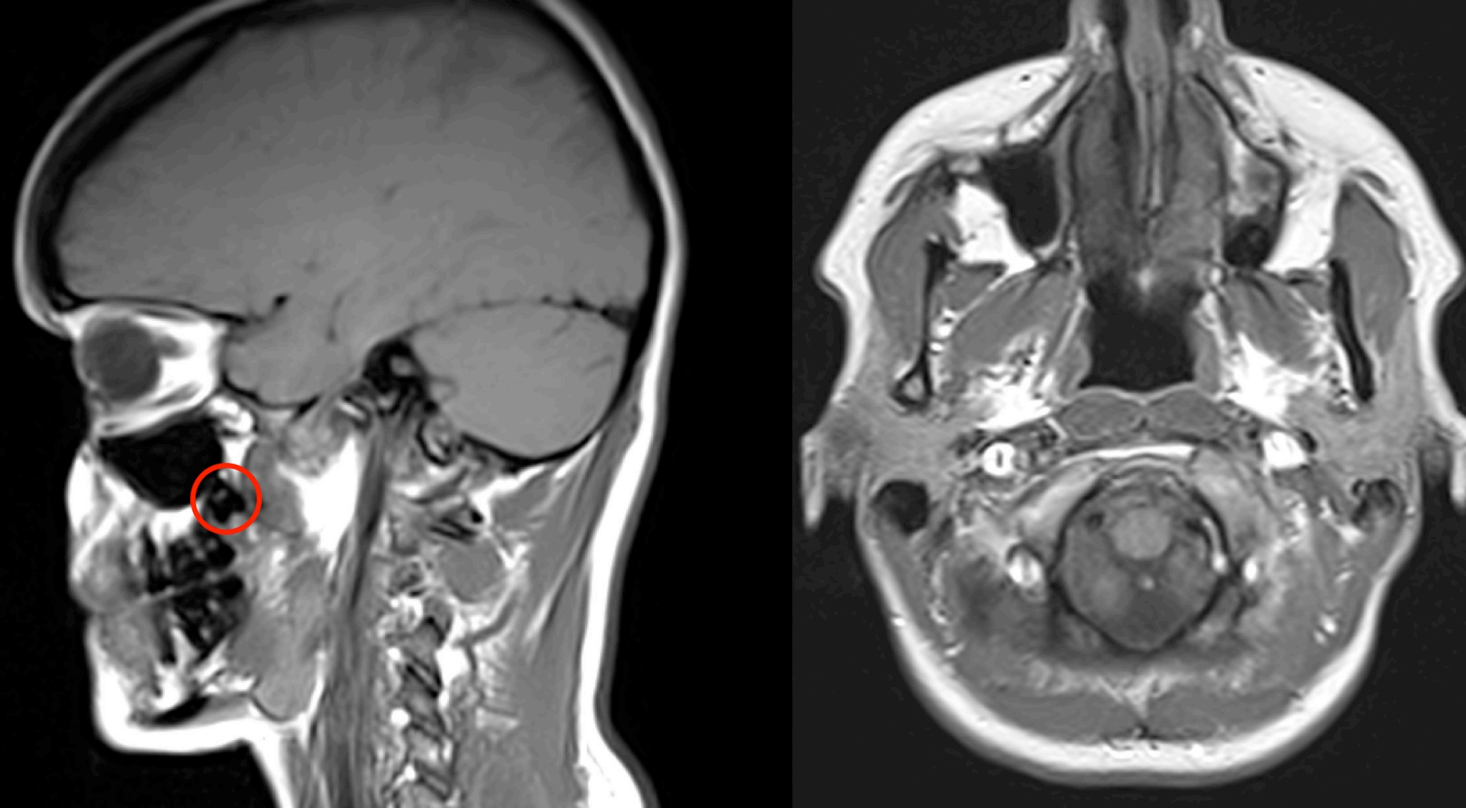
Detection of impacted third molars. Third molars were detected using the cross-referencing tool of OsiriX.

All MRI examinations were performed using a 1.5-T magnetic resonance scanner (Magnetom Avanto; Siemens MedicalSystems, Erlangen, Germany). The imaging protocol is described in detail elsewhere [43]. For evaluation of third molars transversal T1-weighted turbo spin echo images (TE: 11ms, TR: 587ms, slice thickness: 4 mm, matrix: 256 x 256) and sagittal T1-weighted turbo spin echo images (TE: 120ms, TR: 6760ms, slice thickness: 4 mm, matrix: 448 x 448), were used, both of which included the maxilla and the mandible. Additionally, coronal oriented T2-weighted fat suppressed images (TR 4891 ms, TE 670 ms, TI 160 ms, slice thickness 5 mm) were available. For third molar analysis, MR images were transferred to a working station (iMac, OSX) with OsiriX v.3.8.1 software (Pixmeo, Geneva, Switzerland). Two trained dentists analyzed third molars. Inter-observer agreement for third molars for the maxilla was a little higher (kappa: 0.90–0.94) than for the mandible (kappa: 0.81–0.83). In cases with disagreement, the assessment of the dentist with more radiological experience and more involvement in the development of the protocol was used. Image analysis of third molars followed a predefined algorithm. Axial and sagittal images were displayed simultaneously and the cross-referencing tool of OsiriX was used for exact anatomical correlations. Sagittal images were zoomed until an adequate analysis of the third molars was possible (Fig 1). On axial images, the number of teeth was counted for each quadrant and the molars were identified. A three-category third molar variable was defined according to presence and impaction of third molars: 1. Third molar not present; 2. Erupted third molar; 3. Impacted third molar. Existing third molars of the mandible and the maxilla were categorized using the Pell and Gregory classification [44]. Level A: The occlusal plane of the third molar was on the same level as the occlusal plane of the second molar; Level B: The occlusal plane of the third molar was below the occlusal plane of the second molar, but above the cervical line of the second molar; Level C: The occlusal plane of the third molar was below the cervical line of the second molar. Third molars of Level A and Level B were classified as erupted. Level C was classified as impacted. Upper third molars were evaluated as localized in the maxillary sinus if 50% or more of an impacted third molar was located ibidem [45].

### Craniofacial measurements

For craniofacial measurement axial T1-weighted head scans were used (ultra-fast gradient echo sequence, imaging parameters: repetition time of 1900 ms; echo time of 3.37 ms; flip angle of 15^o^; matrix of 176 × 256 × 176; voxel size of 1 × 1 × 1 mm). The post-processing of the axial T1-weighted sequences comprised multi-planar reconstruction (MPR) with 1 mm slice thickness for further image interpretation. Three-dimensional coordinates for each image were calculated from the DICOM headers, which were based on the MRI scanner coordinates. Osirix determined the coordinates (x, y, z) for each voxel and converted the actual calculated size of voxels to millimeters.

**Fig 2:**
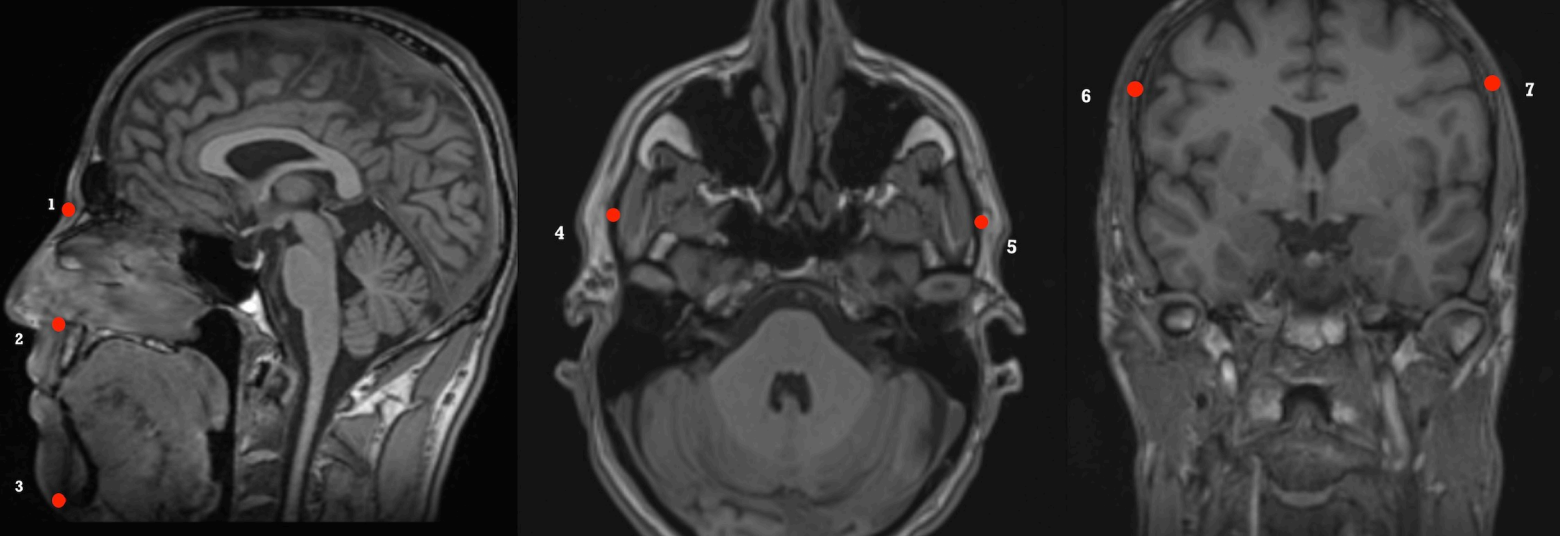
Landmarks were detected using multi planar reconstruction (MPR). 1. Nasion, 2. Anterior nasal spine, 3. Menton, 4. Left Zygion, 5. Right Zygion, 6. Left Eurion, 7. Right Eurion.

Three-dimensional (3D) analysis of the craniofacial morphology was performed with a selected set of predefined landmarks and the predefined distances were subsequently calculated. The following inter-osseous landmarks were included in the analysis: Anterior nasal spine (ANS), Nasion (N), Zygion (Zy), Eurion (Eu) and Menton (Me) (Fig 2). Afterwards, the following distances were calculated as Euclidean distances: Eu-Eu representing the maximal cranial width, Zy-Zy representing facial width, N- ANS representing upper facial height, ANS-Me representing lower facial height and N-Me representing total facial height. The facial index was used to assess facial morphology in five categories, ranging from broad to long faces: $facial\;index=\frac{total\;facial\;height}{face\;width}\times 100$ [33].

Craniofacial MRI measurements of the anatomical landmarks were standardized as follows: First, the position of each landmark on the MRI images was defined through a standardized operating procedure (SOP), second the reproducibility and reliability of each landmark coordinates on the images were assessed, and third the absolute and relative measurement error on the MRI images that were used to detect the landmarks was evaluated.

Three examiners performed landmark detection and measurements over a period of 6 months. All examiners were dentists and were previously trained in the use of Osirix software and craniofacial landmark identification. For investigator blinding, the images were identified by code and analyzed anonymously in random order. The examiners followed the anatomical definition of each landmark. Inter- and intra-examiner reproducibility were assessed, using intra-class correlation coefficients (ICC). The inter-reader agreement was very good and all ICCs laid over 0.9 (S2 Table) [27]. The distances between landmarks were only calculated after their landmarks showed good reproducibility. We also examined the measurement error of the calculated distances in the context of MRI imaging.

The applicability and reproducibility of the landmark detection and measurement methods were established and published previously on the same cohort (SHIP-2) [26]. Furthermore, methods and recommendations to reduce measurement error and observer error on MRI images were established in a previous study and were applied accordingly [33].

### Statistical methods

Stratified by third molar status (missing, erupted or at least one third molar impacted) continuous data was reported as median, 25th, and 75th percentile, and categorical data as absolute numbers and percentages. Facial Measurements were associated with the number of (impacted) third molars by negative binomial regression adjusted for age and sex and are reported as rate ratio and 95% confidence interval. Facial Measurements were also associated with third molar status by multinomial logistic regression models with erupted third molar as reference outcome category adjusted for age and sex. For these analyses two strategies were followed. In the first analysis, we aggregated the third molar data to one observation for each participant using the three levels (1) no third molar (2) erupted third molar, and (3) at least one third molar impacted. In the second analysis, we used four observations from each participant indicating the third molar status in each quadrant of the jaw. This data was analyzed by a mixed effect multinomial logistic regression models with random intercept using the gsem command in Stata 14.2 (Stata Corporation, College Station, TX, USA). Since we assume a physiological association of face morphology with the third molar status, we did not report results accounting for missing data. However, introducing inverse probability weights to account for missing at random did not change the results significantly (data not shown). Our study followed the STROBE criteria for Strengthening the Reporting of Observational Studies in Epidemiology. Additionally, the analyses were stratified for maxilla and mandible. In all analyses a p<0.05 was considered as statistically significant.

## Results

**Fig 3:**
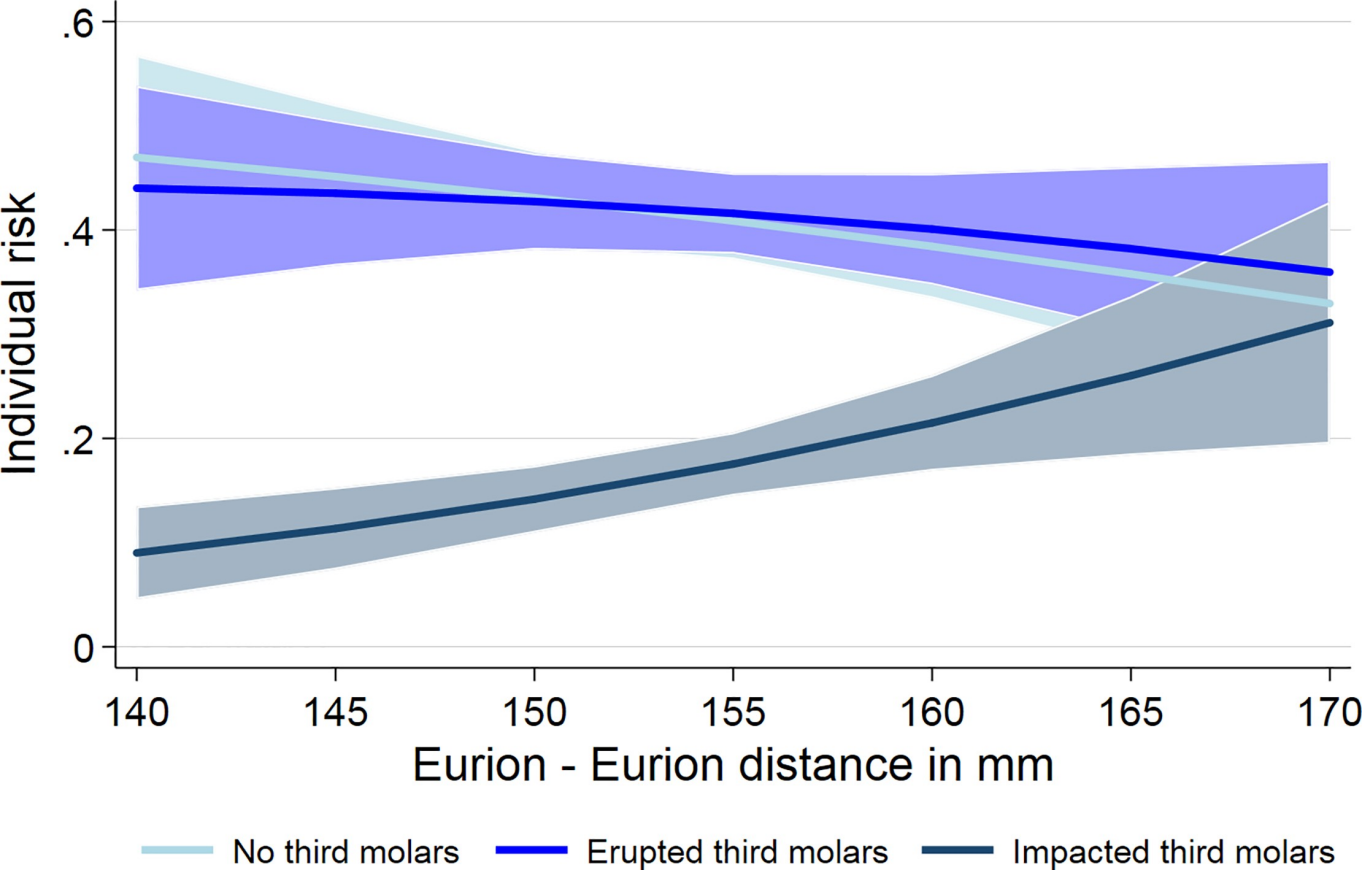
Association of third molar status with Eurion–Eurion distance. An increase of Eurion–Eurion distance by 1 mm led to a significant increase of the number of impacted third molars by 7%.

Of the 619 individuals, 254 had no third molars, 258 erupted third molars, and 107 at least one impacted third molar (Table 1). For the maximal cranial width (Eurion–Eurion distance) we observed significant associations with third molar status after adjustment for age and sex (Table 2). Already with an increasing distance of 1 mm of Eurion–Eurion distance the number of impacted third molars increased significantly by 7% (Table 2, Fig 3). The maximal cranial width was positively associated with impacted third molars in the aggregated third molar data as well as in the mixed model (Tables 2 and 3). This association was observed for impacted third molars in maxilla and mandible and was more pronounced in the mandible. An increase of 1 mm in Eurion–Eurion distance was associated with a 13% increased risk for at least one impacted third molar in the mandible (RR: 1.130; 95% confidence interval 1.043; 1.224) (Table 3). In the maxilla the RR for the Eurion–Eurion distance was 1.094 (95% confidence interval 1.031; 1.161) indicating a 9% increased risk for at least one impacted third molar by an increase of 1 mm in Eurion–Eurion distance. Combining third molar status of maxilla and mandible, no significant associations were observed for the facial index and the other facial distances with third molar status. In the mandible, however, we observed significant inverse associations between the Nasion–Menton distance and impacted third molars in the aggregated data and in the mixed model. By a decrease of 1 mm in Nasion–Menton distance the risk for an impacted third molar in the mandible increased by 8.9% in the mixed model. In the aggregated data only, there was a significant inverse association between the facial index and impacted third molars. No associations were found in the sagittal dimensions.

**Table 1:** Characteristics of the study population stratified by third molar status.

	No third molars (N = 254)	Erupted third molars (N = 258)	At least one impacted third molar (N = 107)
**Age; years**	65 (55; 71) 62 (12)	54 (47; 62) 54 (11)	53 (44; 65) 54 (13)
**Males**	123 (48.4%)	127 (49.2%)	61 (57.0%)
**Facial index**	88.6 (84.5; 92.4) 88.2 (6.1)	88.7 (84.6; 92.5) 88.7 (5.9)	88.2 (84.3; 91.4) 88.1 (5.0)
**Eurion–Eurion; mm**	153.6 (147.9; 159.2) 153.9 (7.8)	153.4 (147.8; 159.0) 153.6 (8.0)	156.2 (149.6; 162.3) 156.2 (8.6)
**Zygion–Zygion; mm**	127.3 (123.0; 133.0) 128.1 (6.2)	127.2 (124.0; 132.1) 127.9 (6.4)	129.1 (124.2; 132.1) 128.6 (6.0)
**Nasion–ANS; mm**	47.1 (43.7; 50.1) 47.5 (4.7)	46.5 (43.2; 50.0) 46.8 (4.4)	46.9 (44.1; 50.6) 47.4 (4.2)
**Nasion–Menton; mm**	112.7 (106.2; 119.3) 112.9 (8.9)	113.5 (106.8; 118.9) 113.4 (8.7)	111.1 (108.3; 118.7) 113.3 (7.5)
**ANS–Menton; mm**	64.6 (59.1; 69.5) 64.5 (7.7)	65.5 (60.8; 69.8) 65.7 (7.4)	63.6 (59.5; 69.0) 64.8 (6.5)
**Nasion–Sella; mm**	77.2 (74.2; 80.1) 77.0 (4.2)	77.0 (74.3; 80.8) 77.5 (4.6)	77.5 (74.8; 80.3) 77.6 (4.4)
**Sella–Basion; mm**	63.2 (59.9; 66.9) 63.6 (5.5)	63.5 (60.4; 67.4) 64.3 (5.6)	63.3 (59.9; 66.8) 63.2 (4.9)
**Nasion–Basion; mm**	122.6 (118.2; 127.0) 122.6 (6.3)	123.2 (118.3; 128.1) 123.7 (7.0)	123.2 (119.6; 127.6) 123.4 (6.1)
**Basion–ANS; mm**	101.1 (96.4; 104.6) 100.7 (6.2)	101.5 (97.2; 106.7) 102.2 (5.8)	101.7 (97.7; 106.8) 101.9 (5.8)

Data are expressed as median, 25^th^, and 75^th^ percentile (first line) and mean and standard deviation (second line) for continuous variables and as absolute number and percentage for dichotomous variables

**Table 2:** Association between face morphology and third molars adjusted for age and sex.

	Number of third molars^a^ RR (95%-CI)	Number of impacted third molars^a^ RR (95%-CI)	No third molars^b^ RR (95%-CI)	Erupted third molars^b^ RR (95%-CI)	At least one impacted third molar^b^ RR (95%-CI)
**Facial index**	0.997 (0.983; 1.011)	0.968 (0.931; 1.007)	0.989 (0.959; 1.021)	Reference	0.978 (0.940; 1.018)
**Eurion–Eurion; mm**	1.016 (1.003; 1.030)*	1.069 (1.035; 1.105)*	0.992 (0.962; 1.022)	Reference	1.050 (1.011; 1.090)*
**Zygion–Zygion; mm**	1.007 (0.989; 1.025)	1.028 (0.797; 1.080)	0.991 (0.951; 1.032)	Reference	1.001 (0.952; 1.053)
**Nasion–ANS; mm**	0.997 (0.977; 1.018)	1.031 (0.976; 1.089)	1.029 (0.984; 1.076)	Reference	1.022 (0.965; 1.083)
**Nasion–Menton; mm**	1.000 (0.988; 1.011)	0.983 (0.953; 1.014)	0.988 (0.963; 1.014)	Reference	0.982 (0.951; 1.014)
**ANS–Menton; mm**	1.000 (0.988; 1.012)	0.972 (0.941; 1.004)	0.979 (0.954; 1.006)	Reference	0.972 (0.940; 1.005)
**Nasion–Sella; mm**	0.982 (0.959; 1.004)	0.966 (0.907; 1.028)	1.017 (0.965; 1.072)	Reference	0.969 (0.908; 1.034)
**Sella–Basion; mm**	0.993 (0.976; 1.009)	0.958 (0.914; 1.004)	0.973 (0.939; 1.009)	Reference	0.944 (0.900; 0.991)*
**Nasion–Basion; mm**	0.995 (0.979; 1.011)	0.979 (0.936; 1.023)	0.983 (0.948; 1.019)	Reference	0.959 (0.915; 1.004)
**Basion–ANS; mm**	1.006 (0.991; 1.020)	1.006 (0.967; 1.047)	0.971 (0.939; 1.004)	Reference	0.973 (0.933; 1.014)
**Maxilla**					
**Facial index**	1.005 (0.986; 1.024)	0.990 (0.951; 1.032)	0.996 (0.963; 1.030)	Reference	0.991 (0.943; 1.040)
**Eurion–Eurion; mm**	1.015 (0.998; 1.032)	1.070 (1.033; 1.109)*	1.001 (0.970; 1.034)	Reference	1.069 (1.021; 1.119)*
**Zygion–Zygion; mm**	1.001 (0.978; 1.025)	1.033 (0.982; 1.087)	1.015 (0.972; 1.059)	Reference	1.036 (0.974; 1.102)
**Nasion–ANS; mm**	1.007 (0.980; 1.034)	1.042 (0.984; 1.102)	1.026 (0.976; 1.078)	Reference	1.071 (0.999; 1.147)
**Nasion–Menton; mm**	1.004 (0.989; 1.019)	1.004 (0.972; 1.037)	1.002 (0.975; 1.030)	Reference	1.005 (0.967; 1.045)
**ANS–Menton; mm**	0.999 (0.984; 1.015)	0.980 (0.948; 1.014)	0.993 (0.965; 1.021)	Reference	0.969 (0.930; 1.009)
**Nasion–Sella; mm**	0.976 (0.947; 1.006)	0.967 (0.904; 1.033)	1.034 (0.978; 1.093)	Reference	0.979 (0.904; 1.060)
**Sella–Basion; mm**	0.984 (0.962; 1.006)	0.963 (0.916; 1.012)	1.021 (0.981; 1.062)	Reference	0.962 (0.907; 1.021)
**Nasion–Basion; mm**	0.990 (0.969; 1.011)	0.978 (0.933; 1.026)	1.011 (0.973; 1.051)	Reference	0.972 (0.918; 1.029)
**Basion–ANS; mm**	1.005 (0.985; 1.024)	1.013 (0.970; 1.057)	0.990 (0.956; 1.025)	Reference	0.997 (0.948; 1.048)
**Mandible**					
**Facial index**	0.992 (0.976; 1.008)	0.943 (0.894; 0.995)*	0.990 (0.960; 1.020)	Reference	0.943 (0.894; 0.995)*
**Eurion–Eurion; mm**	1.015 (1.001; 1.030)*	1.071 (1.023; 1.121)*	0.992 (0.963; 1.021)	Reference	1.058 (1.008; 1.111)*
**Zygion–Zygion; mm**	1.009 (0.989; 1.030)	1.026 (0.957; 1.100)	0.988 (0.950; 1.028)	Reference	1.006 (0.943; 1.075)
**Nasion–ANS; mm**	0.989 (0.965; 1.012)	0.991 (0.917; 1.070)	1.024 (0.980; 1.069)	Reference	0.981 (0.907; 1.061)
**Nasion–Menton; mm**	0.997 (0.984; 1.010)	0.959 (0.919; 1.002)	0.988 (0.964; 1.012)	Reference	0.953 (0.913; 0.996)*
**ANS–Menton; mm**	1.002 (0.989; 1.015)	0.974 (0.932; 1.019)	0.980 (0.955; 1.005)	Reference	0.978 (0.929; 1.015)
**Nasion–Sella; mm**	0.986 (0.962; 1.013)	0.977 (0.896; 1.065)	1.038 (0.987; 1.092)	Reference	0.995 (0.914; 1.084)
**Sella–Basion; mm**	1.000 (0.981; 1.019)	0.964 (0.905; 1.027)	0.989 (0.956; 1.024)	Reference	0.978 (0.919; 1.041)
**Nasion–Basion; mm**	0.999 (0.981; 1.018)	0.989 (0.932; 1.050)	1.004 (0.969; 1.039)	Reference	0.987 (0.929; 1.049)
**Basion–ANS; mm**	1.006 (0.990; 1.023)	0.998 (0.947; 1.052)	0.986 (0.955; 1.018)	Reference	0.973 (0.922; 1.028)

**a** Negative binomial regression adjusted for age and sex

**b** Multinomial logistic regression adjusted for age and sex

* p<0.05; RR rate ratio; CI confidence interval

**Table 3:** Multilevel multinomial logistic regression for the association between face morphology and third molars adjusted for age and sex.

	No third molar^a^ RR (95%-CI)	Erupted third molar^a^ RR (95%-CI)	Impacted third molar^a^ RR (95%-CI)
**Facial index**	0.994 (0.962; 1.026)	Reference	0.957 (0.906; 1.010)
**Eurion–Eurion; mm**	0.982 (0.953; 1.013)	Reference	1.079 (1.028; 1.132)*
**Zygion–Zygion; mm**	0.987 (0.948; 1.028)	Reference	1.017 (0.952; 1.086)
**Nasion–ANS; mm**	1.015 (0.969; 1.063)	Reference	1.028 (0.953; 1.108)
**Nasion–Menton; mm**	0.990 (0.965; 1.016)	Reference	0.970 (0.929; 1.014)
**ANS–Menton; mm**	0.988 (0.962; 1.015)	Reference	0.960 (0.918; 1.004)
**Nasion–Sella; mm**	1.035 (0.982; 1.091)	Reference	0.965 (0.885; 1.052)
**Sella–Basion; mm**	1.005 (0.968; 1.043)	Reference	0.943 (0.883; 1.006)
**Nasion–Basion; mm**	1.002 (0.966; 1.039)	Reference	0.964 (0.907; 1.025)
**Basion–ANS; mm**	0.982 (0.950; 1.015)	Reference	0.985 (0.932; 1.040)
**Maxilla**			
**Facial index**	0.979 (0.926; 1.035)	Reference	0.974 (0.912; 1.041g)
**Eurion–Eurion; mm**	0.995 (0.945; 1.048)	Reference	1.094 (1.031; 1.161)*
**Zygion–Zygion; mm**	0.997 (0.931; 1.069)	Reference	1.043 (0.964; 1.128)
**Nasion–ANS; mm**	0.994 (0.919; 1.075)	Reference	1.059 (0.966; 1.160)
**Nasion–Menton; mm**	0.982 (0.939; 1.027)	Reference	0.996 (0.944; 1.049)
**ANS–Menton; mm**	0.991 (0.946; 1.037)	Reference	0.964 (0.912; 1.018)
**Nasion–Sella; mm**	1.070 (0.977; 1.171)	Reference	0.985 (0.888; 1.094)
**Sella–Basion; mm**	1.046 (0.980; 1.116)	Reference	0.965 (0.890; 1.046)
**Nasion–Basion; mm**	1.023 (0.960; 1.089)	Reference	0.977 (0.907; 1.053)
**Basion–ANS; mm**	0.984 (0.929; 1.042)	Reference	1.005 (0.940; 1.076)
**Mandible**			
**Facial index**	1.005 (0.966; 1.046)	Reference	0.897 (0.794; 1.014)
**Eurion–Eurion; mm**	0.971 (0.935; 1.008)	Reference	1.130 (1.043; 1.224)*
**Zygion–Zygion; mm**	0.975 (0.928; 1.024)	Reference	0.984 (0.892; 1.085)
**Nasion–ANS; mm**	1.029 (0.972; 1.089)	Reference	0.955 (0.821; 1.112)
**Nasion–Menton; mm**	0.998 (0.967; 1.031)	Reference	0.911 (0.844; 0.982)*
**ANS–Menton; mm**	0.988 (0.956; 1.022)	Reference	0.941 (0.844; 1.049)
**Nasion–Sella; mm**	1.033 (0.967; 1.103)	Reference	0.943 (0.810; 1.097)
**Sella–Basion; mm**	0.992 (0.948; 1.039)	Reference	0.920 (0.814; 1.039)
**Nasion–Basion; mm**	1.000 (0.955; 1.046)	Reference	0.969 (0.942; 0.997)*
**Basion–ANS; mm**	0.982 (0.942; 1.023)	Reference	0.989 (0.903; 1.084)

a Multilevel multinomial logistic regression adjusted for age and sex

* p<0.05; RR rate ratio; CI confidence interval

## Discussion

In our European descent population, we discovered a positive association between the maximal cranial width measured as Eurion–Eurion distance and the impaction of third molars, i.e. the broader the cranium the higher the risk to have an impacted third molar. Participants with a 1 mm increased maximal cranial width had a 9% increased risk for an impacted third molar. For a two mm increase the risk for an impacted third molar would already increase to 18% (Rate ratio = 1.09*1.09 = 1.18). The observed association was more pronounced in the mandible. Furthermore, a decrease of the anterior facial height (Nasion–Menton) and a decreased facial index was associated with an increased risk for an impacted third molar in the mandible.

Genetic differences in ethnicities manifested by different craniofacial morphologies [46] may be a possible explanation for the varying impaction proportions worldwide. Likewise, different individual genetic predispositions were discussed to affect third molar impaction [47]. The association of the maximal cranial width with impacted third molars may be a possible manifestation of a genetic influence in the population analyzed in this study. Dental eruption sequence may be conserved phylogenetically [8]. Monson et al. [8] described in a sample of 194 individuals representing 21 primate genera, that dental eruption sequence would be conserved phylogenetically in primates and dental eruption sequence to be associated with body and brain size. This might be in line with the association of the maximal cranial width with impacted third molars in our study. Similar to our findings, in craniometric landmark measurements of 131 archaeological skulls from 6 populations rotated, third molars seem to be more frequent in populations with low broad skulls [48].

The skeletal facial type has been associated with third molar impaction [10, 15, 16]. Our findings are consistent with studies describing a higher prevalence of impacted third molars in individuals with reduced facial height (brachyfacial type) [15, 16, 40]. Our findings stand in contrast with findings describing higher impaction proportions in individuals with an increased anterior facial height (dolichofacial type) [10]. The partially contradicting results in the lateral perspective may be caused by selection bias, since craniofacial morphology and third molars were assessed by X-ray based radiographs in patient samples [3, 10, 15, 16]. These partly contradicting results may also be caused by the limitations of 2D assessment modalities. Assessment of craniofacial morphology in orthodontic patients is normally conducted with orthopantomograms, photometric analysis and cephalometric radiographs [17, 18]. There is a debate whether 3D methods are more informative than conventional 2D methods and further research is required to clarify it. [49] Three-dimensional imaging methods have been used for analysis of craniofacial morphology and assessing treatment outcome in cleft lip and palate patients [49]. MRI is considered suitable for accurate and reproducible 3D measurements of the craniofacial skeleton allowing a more extended analysis of the craniofacial complex than 2D cephalometric radiography [20, 50], Nonetheless, some previous studies [51]found no clear evidence for better reliability of craniofacial landmark analysis than with the conventional cephalometric methods.

However, independently of the technical aspects of different imaging modalities, the main focus of conventional cephalometric analysis using lateral cephalogramms lies on analysis of the lateral perspective of the craniofacial morphology. The frontal perspective is regularly assessed with photometric analysis of the soft tissues. In the photometric analysis the bizygomatic distance is used as a common distance for evaluation of the facial width [52]. The application of 3D MRI application allows for analysis of new perspectives of craniofacial image analysis without additional exposure to radiation and for assessment of bone and the surrounding soft tissue [25]. Assessment of the facial width defined by the eurion–eurion distance without soft tissue coverage is possible with the application of 3D MRI-analysis in a population based sample and the association with impacted third molars was discovered. In a heterogenic population-based sample the discovered effects of 18% increased impaction risk with an increase of 2 mm facial width could be clinically relevant for orthodontic or maxillofacial surgery treatment assessment. The discovered associations need to be specified and clarified in further advanced analysis like geometric morphometry.

The discovered association of impacted third molars with the heads width may also be linked to masticatory benefits of food processing changes in evolution followed by changes in craniofacial morphology. Changes in nutrition during evolution may have resulted in a reduction of the jaw size [53]. Consequently, the lack of a coarse, abrasive diet might be the major cause for impacted teeth in modern civilization [54]. Third molars impaction may also follow the origins of cooking and other food-softening behaviors in the way of a genetic drift [55] and be linked in an unknown way to the maximal cranial width. Potentially these factors can be responsible for the observed associations between craniofacial morphology and impacted third molars.

To the best of our knowledge no studies have evaluated the relation between third molar impaction and skeletal pattern using MRI in a population-based cohort. This hampers the comparison of our results with those previously obtained.

Our study suggests that cranial structures, craniometric points and distances can be sufficiently evaluated with a standard cerebral MRI procedure in good agreement with classical anthropometrical evaluation [28]. Therefore, our study can be considered as hypothesis generating study. Because of the cross-sectional design of our study, it is not possible to draw causal conclusions. The strengths of our study are, that SHIP is a large study with high level of quality assurance, the use of non X-ray based MRI and the strict adherence to standardization of examination methods and data management [29]. The need of preventing unnecessary radiographic diagnostics resulting in sampling bias seems to be a major limitation for analysis of impacted third molars even in large meta-analyses [3]. However, there are limitations in our study that must be considered. First, due to the study design, not every radiological orthodontic landmark could have been evaluated, which may lead to a reduced comparability to existing studies that used direct measurements on patients or skull remains. Second, the federal committee of MRI (Gemeinsamer Bundesausschuss) described a slice thickness of 5 mm as suitable for head and neck diagnostics [56]. In our study the slice thickness was 4 mm and the gap between two slices was 10%. Due to this slice thickness partially erupted third molars and impacted third molars may have been misclassified. This could have affected our results. A 3D-MR data set with dental reconstructions perpendicular to the mandible and maxilla may have improved MR diagnostics; otherwise the observer agreement in our study was very good. Third, assessment of third molar agenesis was limited in our study, because we had no information whether third molars were removed or not developed. According to a large meta-analysis the prevalence of third molars agenesis worldwide was approximately 20% [57]. A relationship between third molars impaction and agenesis is discussed but they may also be two unrelated phenomena with different developmental origins [3]. We tried to partially compensate this methodological limitation by using erupted third molars as reference in the analysis. However, even if the information about agenesis or removal would have been available, there is still a great variation among general dental practitioners for decision-making of third molars removal [58]. Likewise, a vivid discussion about the risk ratio between need and harm for removal of third molars exists [59–62]. In addition, access to medical/dental treatment, differences in insurance systems, surgical specialization and payment by general health insurance system may contribute to this variation in decision-making of third molars removal [59] [63].

Our results also show a tendency that participants with a short face and participants with an increased maximal cranial width have an increased risk for an impacted third molar in the mandible. A combination of the discovered effects may intensify the risk for third molars impaction especially in the mandible. Our results could support Behbehani et al. [40] who aimed to establish a predictive model for mandibular third-molar impaction before and after orthodontic treatment and tried to determine the optimal timing for removal.

In conclusion, individuals with an increased maximal cranial width and decreased anterior facial height have a higher risk for impaction of third molars in the mandible. These findings may be helpful for orthodontic dentists, oral surgeons and oral and maxillofacial surgeons in the decision-making of third molars removal in planning and assessing their treatment. These findings support the necessity of additional analysis of the Eurion—Eurion distance.

### Compliance with ethical standards

Informed consent was obtained from all individual participants included in the study and all investigations were performed in accordance with the Declaration of Helsinki.

## Supporting information

S1 Table STROBE statement—Checklist of items that should be included in reports of *cross-sectional studies*. (DOC)

S2 Table Intraclass correlation coefficients (ICC) for all coordinates used for landmark identification. (DOCX)
