# Supplementary material for: Does craniofacial morphology affect third molars impaction? Results from a population-based study in northeastern Germany
Source: PLoS One. 2019 Nov 22;14(11):e0225444. doi: 10.1371/journal.pone.0225444 (PMC6874347; doi:10.1371/journal.pone.0225444)
Supplement: S2 Table — (DOCX) [file pone.0225444.s002.docx]

**Supplemental Material**

S2 Table Intraclass correlation coefficients (ICC) for all coordinates used for landmark identification.

| Landmark | X Coordinate | Y Coordinate | Z Coordinate |
| --- | --- | --- | --- |
| Nasion | 0.95 (0.90-0.98) | 0.96 (0.77-0.99) | 0.97 (0.86-0.99) |
| Anterior nasal spine | 0.99 (0.98-0.99) | 0.94 (0.88-0.97) | 0.96 (0.71-0.98) |
| Right Eurion | 0.94 (0.86-0.97) | 0.95 (0.83-0.98) | 0.97 (0.85-0.99) |
| Left Eurion | 0.97 (0.94-0.98) | 0.96 (0.81-0.99) | 0.97 (0.86-0.99) |
| Right Zygion | 0.98 (0.91-0.99) | 0.98 (0.96-0.99) | 0.99 (0.99-0.99) |
| Left Zygion | 0.98 (0.93-0.99) | 0.99 (0.98-0.99) | 0.99 (0.99-0.99) |
| Menton | 0.99 (0.97-0.99) | 0.99 (0.96-0.99) | 0.98 (0.93-0.99) |
